# Fast and Facile Synthesis of Cobalt-Doped ZIF-8 and Fe_3_O_4_/MCC/Cobalt-Doped ZIF-8 for the Photodegradation of Organic Dyes under Visible Light

**DOI:** 10.1021/acsomega.4c06142

**Published:** 2024-12-07

**Authors:** Amin Mehrehjedy, Piyush Kumar, Zachary Ahmad, Penelope Jankoski, Anuraj S. Kshirsagar, Jason D. Azoulay, Xuyang He, Mahesh K. Gangishetty, Tristan D. Clemons, Xiaodan Gu, Wujian Miao, Song Guo

**Affiliations:** † Department of Chemistry and Biochemistry, School of Mathematics and Natural Sciences, University of Southern Mississippi, Hattiesburg, Mississippi 39406, United States; ‡ School of Polymer Science and Engineering, The University of Southern Mississippi, Hattiesburg, Mississippi 39406, United States; § Department of Chemistry, Mississippi State University, Starkville, Mississippi 39762, United States; ∥ School of Chemistry and Biochemistry and School of Materials Science and Engineering, Georgia Institute of Technology, Atlanta, Georgia 30332, United States; ⊥ School of Criminal Justice, Forensic Science, and Security, The University of Southern Mississippi, Hattiesburg, Mississippi 39406, United States; # Department of Physics and Astronomy, Mississippi State University, Mississippi State, Mississippi 39762, United States

## Abstract

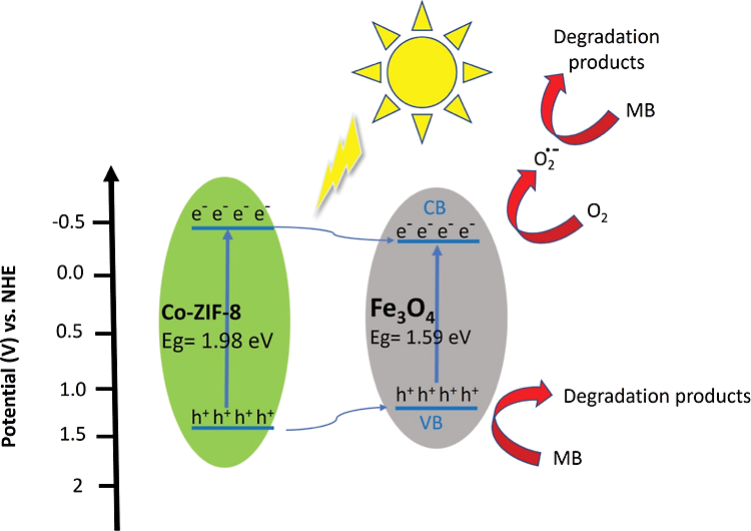

Co-doped ZIF-8 as a water-stable visible light photocatalyst was prepared by using a one-pot, fast, cost-effective, and environmentally friendly method. The band structure of ZIF-8 was tuned through the incorporation of different percentages of cobalt to attain an optimal band gap (*E*_g_) that enables the activation of ZIF-8 under visible light and minimizes the recombination of photogenerated charge carriers. A magnetic composite of Co-doped ZIF-8 was also synthesized to facilitate catalyst recycling and reusability through the application of an external magnetic field. Surface modification of magnetic Fe_3_O_4_ nanoparticles with microcrystalline cellulose (MCC) was used to reduce the level of agglomeration. The photocatalytic activities of Co-doped ZIF-8 (Co-ZIF-8) and Fe_3_O_4_/MCC/Co-ZIF-8 were evaluated for the photodegradation of methylene blue (MB) under visible light irradiation from a 20 W LED source. Co-ZIF-8 showed considerably higher photocatalytic activity than pure ZIF-8, confirming the success of the doping strategy. Both Co20%-ZIF-8 and Fe_3_O_4_/MCC/Co20%-ZIF-8 exhibited similar and remarkable photocatalytic activity under visible light (achieving 97% MB removal). The mechanism of photodegradation of MB by Fe_3_O_4_/MCC/Co20%-ZIF-8 was studied, revealing a first-order degradation kinetics (*k* = 13.78 × 10^–3^ min^–1^), with peroxide and hole species as the predominant active reagents. The magnetic composite successfully displayed recyclability and reusability over multiple cycles with negligible reduction in MB photodegradation efficiency.

## 1 Introduction

Organic dyes are major contributors to water pollution in industrial wastewater. They are often toxic and difficult to decompose.^1^ A green and energy-efficient method for their oxidative degradation is through photocatalytic degradation, which harnesses the power of UV and/or visible light in conjunction with a photocatalyst to generate reactive oxygen species (ROS).^2^ While large band gap semiconductors are active in UV light, whose spectrum accounts for only a small fraction (3–5%) of sunlight.^3^ On the other hand, although photocatalysts with small band gaps are effective under visible light, they often suffer from rapid recombination of photogenerated electron–hole pairs, hindering efficient electron transfer to other species in the aqueous medium and ROS production. Therefore, tuning the band gap and minimizing the charge recombination in the photocatalyst are necessary for efficient visible light activation.^2^

Metal–organic frameworks (MOFs) are a type of inorganic polymer developed recently. They consist of metal cluster centers and organic linkers, which enable various photoinduced charge transfer processes such as metal-to-ligand, ligand-to-metal, and ligand-to-ligand transfers.^4^ MOFs have been utilized in various photocatalytic processes, including selective photooxidation of alcohols,^5^ amine and sulfides,^4^ water splitting,^6^ photoreduction of CO_2_,^7^ and photocatalytic degradation of organic dyes.^8^ MOFs stand out due to their high surface area, tunable cavity, and tailorable chemistry, which contribute to their superior activity compared to other alternatives.^9^ Among the MOFs, zeolitic imidazolate frameworks (ZIFs) form a distinct subclass. ZIFs feature tetrahedrally coordinated transition metal ions connected by imidazolate ligands.^10^ In the case of ZIF-8 and ZIF-67, the respective Zn^2+^ and Co^2+^ metal centers are linked by 2-methylimidazole (Hmim) to form a structure with a SOD (sodalite) topology.^11^ While many MOFs, including ZIF-67, are unstable in aqueous solutions, ZIF-8 exhibits water stability. However, its wide band gap (*E*_g_ = 5.1 eV) restricts its activity to UV light.^12^ A recent report highlights the development of water-stable mixed metal derivatives of ZIF-8 and ZIF-67 with notable photocatalytic activity under visible light.^13^

Another challenge in the photocatalytic degradation of dyes in wastewater is the recycling and reuse of the photocatalyst. Coupling magnetic nanoparticles like iron oxides with the photocatalyst and separating them using a magnet presents a more favorable option compared to time- and energy-consuming processes such as filtration and centrifugation.^14^ Magnetic nanoparticles have low photocatalytic activity due to their small band gap. To address this issue, modification of magnetic nanoparticles with materials possessing a finely tuned band gap, such as metal oxides^15−17^ or MOFs,^18−20^ has been explored to enhance their photocatalytic efficiency, resulting in the development of effective magnetic photocatalysts.^21^ Previously reported methods for fabricating magnetic composites of MOFs, such as the solvothermal method, are time-consuming, taking several hours or even days to complete. These methods require significant amounts of energy, specialized equipment, and the use of organic solvents that are not environmentally friendly.^22,23^ Therefore, a faster, less energy-consuming, low-cost, and environmentally friendly method for the fabrication of magnetic MOFs is necessary for their large-scale production.

Sunlight is available only during the daytime, and its supply can be interrupted by cloudy days or nighttime. To address this challenge, artificial light sources such as sunlight simulators, xenon arc lamps,^13^ mercury-vapor lamp,^10^ and LEDs^2^ have been used as the light sources. Among these options, LEDs are the best alternative to sunlight due to their energy efficiency, durability, long lifespan, and ability to operate on direct current.^24^

In this study, we present a rapid and straightforward method for fabricating Co-ZIF-8s and their magnetic composites in alkaline aqueous solution. The synthesis can be completed in just 20 min and does not require any specialized equipment or heating. The photocatalytic activity of the prepared samples is evaluated through the photodegradation of methylene blue (MB) under visible light. Additionally, the mechanism of photodegradation is investigated by using scavenger tests and Mott–Schottky plots.

## 2 Experimental Section

### 2.1 Synthesis of ZIF-8, ZIF-67, and Co-Doped ZIF-8 and Their Magnetic Composite

All chemicals used were of analytical grade and were used without further purification. ZIF-8, ZIF-67, and mixed metal derivatives were synthesized using stoichiometric concentrated aqueous solutions of metal and ligand in the presence of ammonium hydroxide.^25−27^ Zinc nitrate hexahydrate (Zn(NO_3_)_2_·6H_2_O) and cobalt(II) acetate (Co(OOCCH_3_)_2_·4H_2_O) were dissolved in deionized (DI) water at various ratios to prepare solution A. Additionally, 2-methylimidazole was dissolved in ammonium hydroxide (29% aqueous solution) to prepare solution B. Subsequently, solution B was added dropwise to solution A with a metal-to-ligand ratio of 1:2 under vigorous stirring. The resulting solution was continuously stirred for an additional 20 min to complete the crystallization of the ZIF samples. The precipitations were washed several times to reduce the pH and then dried in an oven at 80 °C overnight.

**Figure 1:**
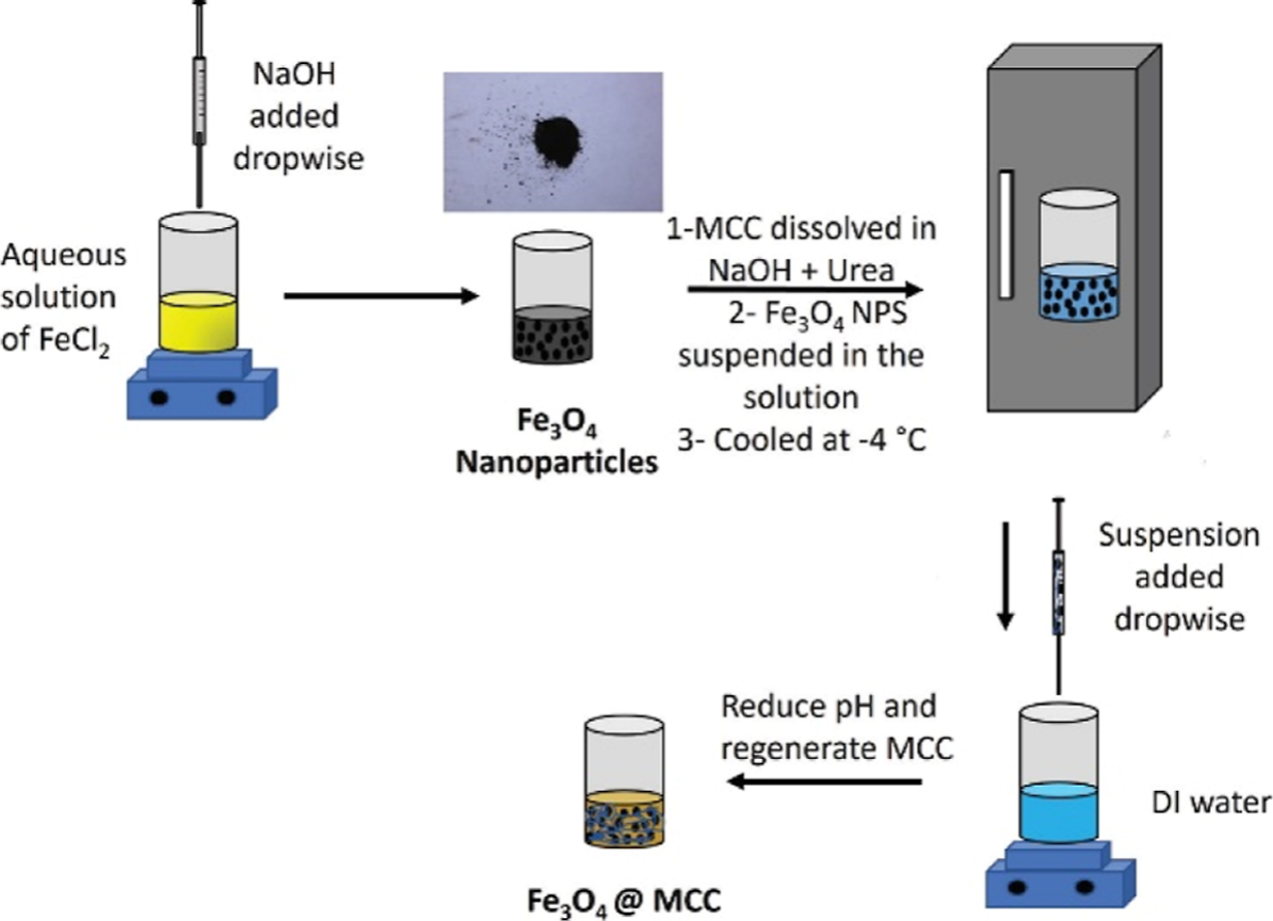
Synthesis of Fe_3_O_4_ and Fe_3_O_4_/ MCC.

Fe_3_O_4_ magnetic nanoparticles were synthesized using a promising method for large-scale production, involving the partial oxidation of Fe^2+^ in an alkali solution. This method is simple, fast, and high yield.^28,29^ As shown in Figure 1, NaOH was added to an aqueous solution of FeCl^2+^ under vigorous stirring to obtain a black magnetic precipitate. The precipitate was then collected using an external magnet, washed multiple times with DI water to reduce the pH, and subsequently dried overnight in an oven to obtain Fe_3_O_4_ nanoparticles.

During composite synthesis, Fe_3_O_4_ magnetic nanoparticles tend to agglomerate, resulting in the formation of two solid phases instead of a uniform composite.^30^ To tackle this issue, microcrystalline cellulose (MCC), a sustainable material, was used to modify the surface of magnetic nanoparticles and reduce agglomeration.^30,31^ Fe_3_O_4_/MCC nanoparticles were prepared by dissolving cellulose in an aqueous solution of urea and NaOH at 0 °C. Subsequently, the magnetic nanoparticles were dispersed in the solution, and MCC crystals were regenerated upon the pH reduction achieved by adding DI water.^32^

**Figure 2:**
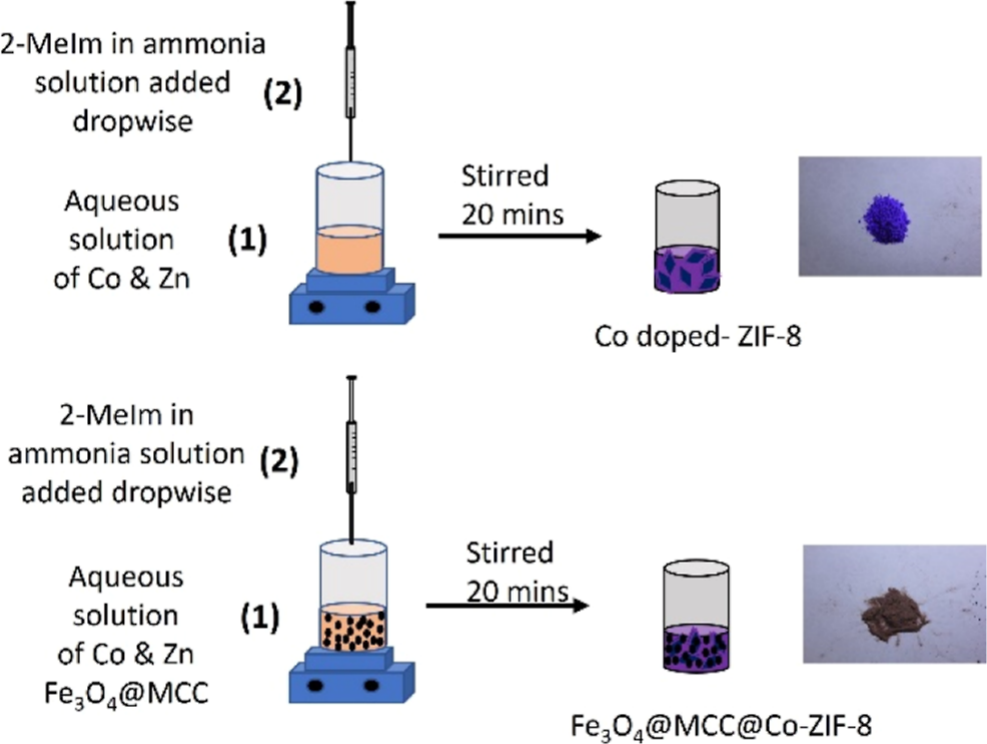
Synthesis of Co-ZIF-8 and Fe_3_O_4_/MCC/Co-ZIF-8.

In the final step, magnetic composites of ZIFs were synthesized by dispersing Fe_3_O_4_/MCC in an aqueous solution of cobalt and zinc salt (Figure 2). Solution B was subsequently added dropwise to the mixture under vigorous stirring, and the solution was stirred for an extra 20 min to complete the crystallization process. The resulting composite was washed several times with DI water to reduce the pH and then dried in an oven overnight.

### 2.2 Characterization

Powder wide-angle X-ray scattering (WAXS) data of the as-prepared samples were collected using a Xenocs Xeuss X-ray scattering beamline with Cu K-alpha (wavelength 0.15418 nm) radiation. The morphology of samples was observed by environmental scanning electron microscopy (ESEM, FEI Quanta) and transmission electron microscopy (TEM, Zeiss EM 900). SEM/EDX (ESEM, FEI Quanta with Thermo System 7 EDS X-ray detectors) was used to study the elemental composition of the samples. Fourier transform infrared spectroscopy (FT-IR) spectra were measured by a Nicolet Summit spectrometer. The ζ-potential was measured using a Zetasizer Nano ZSP instrument (Malvern). Diffuse reflectance spectroscopy (DRS) was performed using a Shimadzu UV-2600i spectrophotometer.

Electrochemical impedance spectroscopy studies were carried out by using an electrochemical analyzer (CH Instruments, CHI660A) in a standard three-electrode system. Slurries were prepared by dispersing the photocatalysts into a solution of H_2_O, ethanol, and Nafion in the ratio of 75:20:5, with Nafion acting as a binder. The working electrode was fabricated by drop-casting 35 μL of slurry onto a carbon-cloth (CC) substrate electrode with a working area of (1 × 0.5) cm^2^. The resulting electrode was then immersed in a 0.5 M Na_2_SO_4_ electrolyte. A saturated Ag/AgCl electrode and platinum mesh were used as the reference and counter electrodes, respectively. Mott–Schottky curves were plotted with the independence-potential model to estimate the band positions of Co-ZIF-8 and the magnetic composite.

### 2.3 Photocatalytic Experiment

The photocatalytic activities of different catalysts were investigated by degrading a model organic dye, MB, in solution under visible light. A water-jacketed photoreactor was equipped with a 20 W white LED lamp as the visible light source. A beaker containing 50 mL of MB solution and 50 mg of the catalyst was placed 20 cm away from the light source during the photodegradation process.

To achieve an adsorption–desorption equilibrium, the catalyst-MB suspension was stirred in the dark for 30 min prior to irradiation. Experiments were carried out at a constant temperature of 25 °C, and samples were collected every 30 min. Changes in the MB concentrations were measured by using a Cary 60 UV–visible spectrometer. The degradation of MB was monitored by analyzing the changes in the absorption spectrum at 665 nm.

## 3 Results and Discussion

### 3.1 Catalyst Characterization

**Figure 3:**
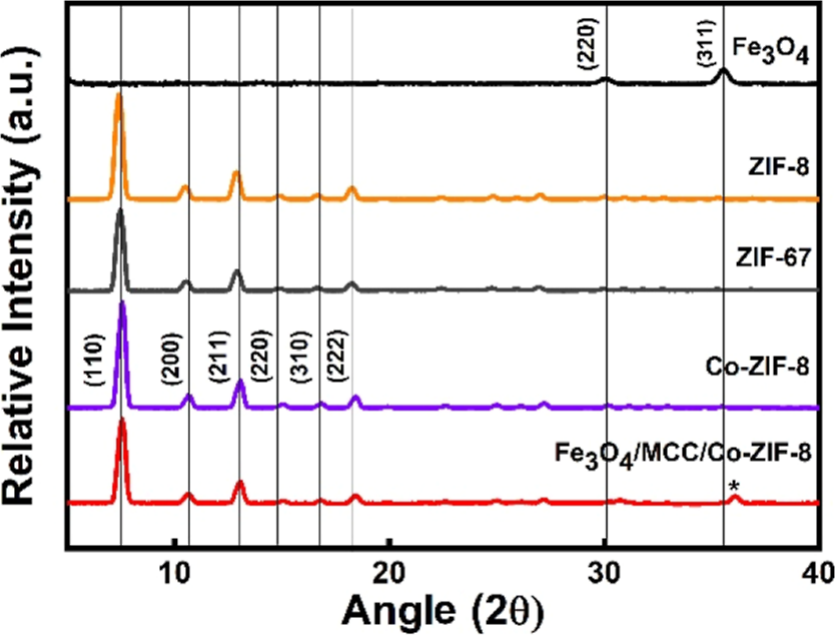
WAXS of ZIF-8, ZIF-67 (intensity × 2), Co-doped ZIF-8, Fe_3_O_4_, and Fe_3_O_4_/MCC/Co20%-ZIF-8 (intensity × 5). * denotes 2θ = 36° arising from the (311) of the magnetic Fe_3_O_4_ nanoparticles.

The WAXS data of the as-prepared ZIF samples, including ZIF-8 and ZIF-67, as shown in Figure 3, are consistent with previous reports.^13^ The prominent peaks observed at 7.4, 10.6, 12.9, 14.9, 16.6, and 18.3° correspond to the (110), (200), (211), (220), (310), and (222) crystallographic planes, respectively. Both ZIF-8 and ZIF-67 exhibit peaks with identical 2θ values, confirming that they are in the same phase. In the cases of Co-ZIF-8 and Fe_3_O_4_/MCC/Co-ZIF-8, the same peaks are observed, confirming the existence of ZIF phases in the composite material. The ZIF peaks in the composite slightly shift to higher 2θ values (0.1°), which could be attributed to the doping effect from cobalt. In the WAXS of Fe_3_O_4_/MCC/Co-ZIF-8, the relative intensities of peaks are lower than those of the pure ZIF samples due to the presence of magnetic nanoparticles. However, the main peaks of Co-ZIF-8 are present in the spectra. The relative intensity of Fe_3_O_4_ peaks is lower than that of Co-ZIF-8, with only the main peak of magnetic nanoparticles (311) observed at 2θ = 36° as marked in the spectra.^33^ The preserved ZIF and Fe_3_O_4_ peaks, as well as the absence of additional peaks, confirm the structural integrity of the synthesized composites.

**Figure 4:**
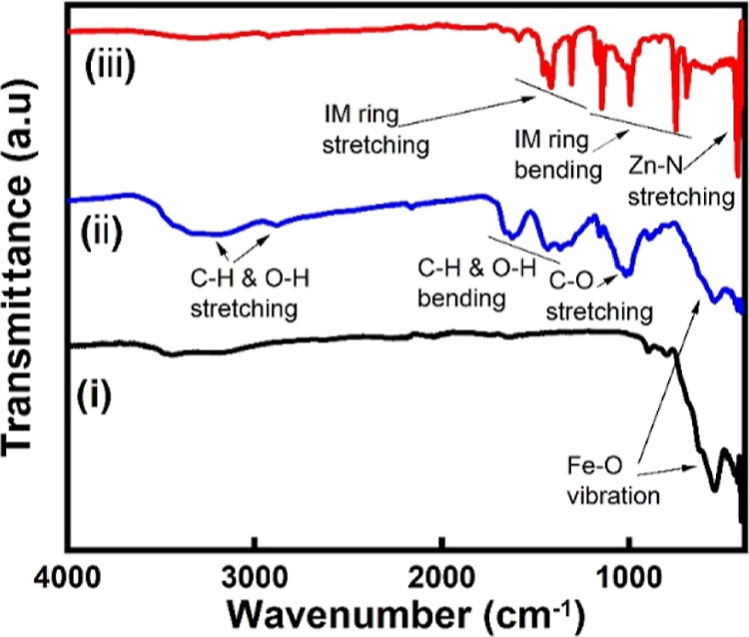
FT-IR of the as-prepared samples. (i) Fe_3_O_4_, (ii) Fe_3_O_4_/MCC, and (iii) Fe_3_O_4_/MCC/Co-ZIF-8.

In Figure 4, the FT-IR spectra of Fe_3_O_4_ exhibited a peak at 580 cm^–1^, which is characteristic of Fe_3_O_4_ and corresponds to the Fe–O vibration. The FT-IR spectra of Fe_3_O_4_/MCC showed peaks at 1047, 1377, 1434, 1648, 2941, and 3426 cm^–1^, attributed to C–O stretching, C–H and O–H bending, and C–H and O–H stretching of cellulose, respectively. All Co-doped ZIF materials, including Co-ZIF-8 and Fe_3_O_4_/MCC/Co-ZIF-8, show characteristic IR peaks of pure ZIF-8 and ZIF-67, as seen in Figure 4. In the case of Co-doped ZIF-8, the band at 421 cm^–1^ can be assigned to the Zn–N stretch mode, and the bands at 500–1350 cm^–1^ are attributed to plane bending. Peaks at 1350–1500 cm^–1^ are attributed to the plane stretching of the imidazole ring. Additionally, a C=N stretch mode was observed at 1584 cm^–1^.^34,35^ The FT-IR data indicate a successful integration between the ZIF, Fe_3_O_4_, and MCC phases within the composite materials.

**Figure 5:**
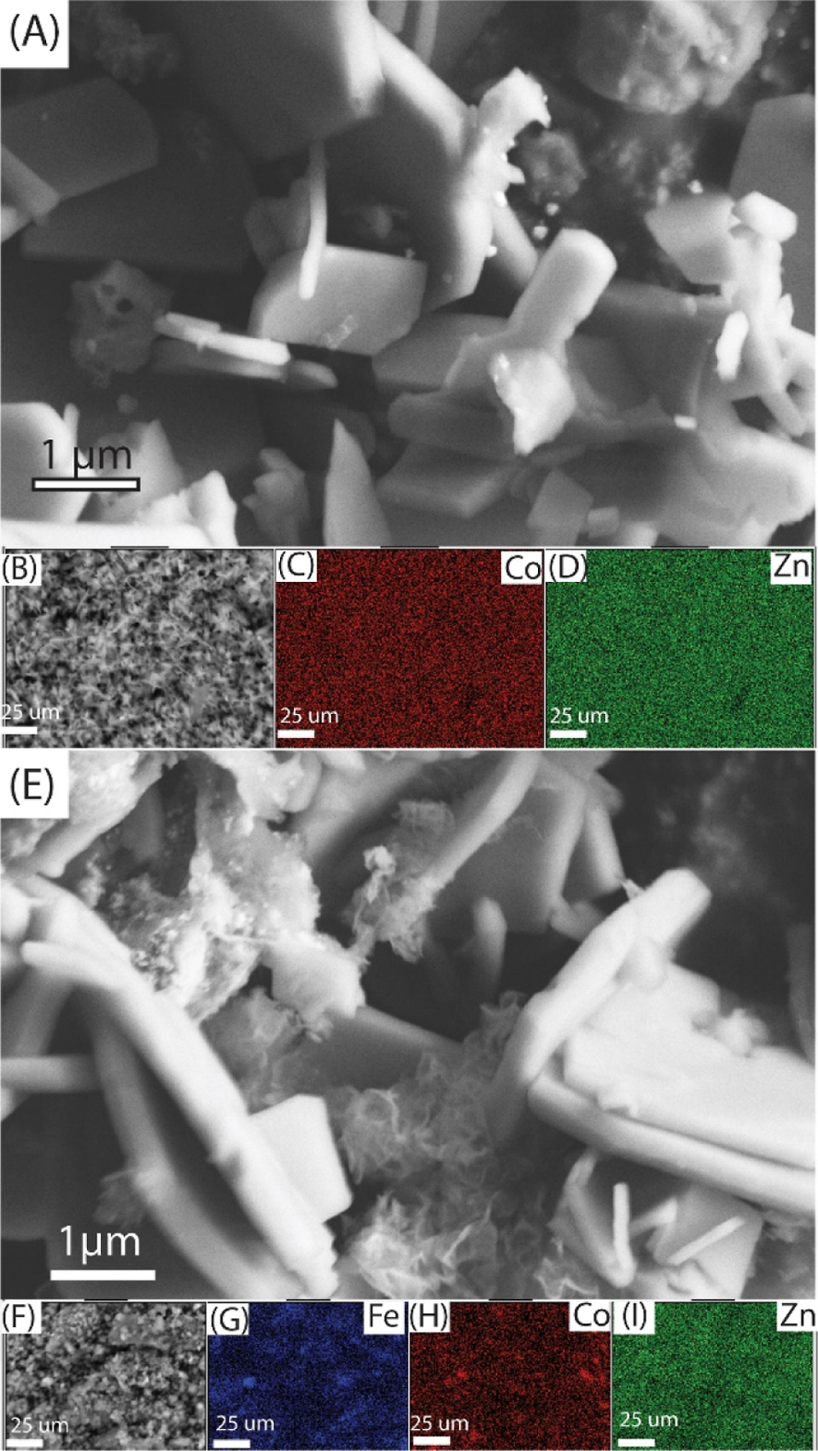
Representative scanning electron micrographs of (A,B) Co-ZIF-8 and (E,F) Fe_3_O_4_/MCC/Co-ZIF-8 and the corresponding elemental mapping of (C,D) Co-ZIF-8 and (G–I) Fe_3_O_4_/MCC/Co-ZIF-8.

The morphologies of the ZIFs and their composites were studied by SEM. The SEM image of Co-ZIF-8 shown in Figure 5A reveals larger plate-like particles compared to the smaller cube-like morphology typically observed for ZIF-8, ZIF-67, and their mixed-metal derivatives reported previously.^13^ The observed morphology could be attributed to the vigorous stirring method used during synthesis,^36^ which is necessary for the formation of a homogeneous magnetic composite of mixed metal ZIF-8. In Figure 5E, the Fe_3_O_4_ nanoparticles can be seen between the plate-like structures of Co-ZIF-8. Figure S2B shows a TEM image of a nanoparticle of Fe_3_O_4_. The TEM image of Co-ZIF-8 (Figure S2A) has a lighter contrast, likely due to the lower mass thickness of ZIF material compared to Fe_3_O_4_. The TEM image of Co-ZIF-8 also shows elongated structures that are consistent with the plate-like structure observed in SEM. For Fe_3_O_4_/MCC/Co-ZIF-8, both Fe_3_O_4_ and Co-ZIF-8 regions are present to form heterojunctions, as shown in Figure S2D.

The SEM/EDX mapping shows an even distribution of both Co and Zn in Co-ZIF-8, indicating successful doping with ZIF-8 (Figure 5C,D). EDX mapping of Fe_3_O_4_/MCC/Co-ZIF-8 (Figure 5G–I) demonstrates a uniform distribution of Co, Zn, and Fe, confirming that Co-ZIF-8 are evenly grown on Fe_3_O_4_/MCC, providing ample interfaces between them. Therefore, it is very likely that a heterojunction is formed between Fe_3_O_4_ and Co-ZIF-8, consistent with SEM, WAXS, and FTIR results.

**Figure 6:**
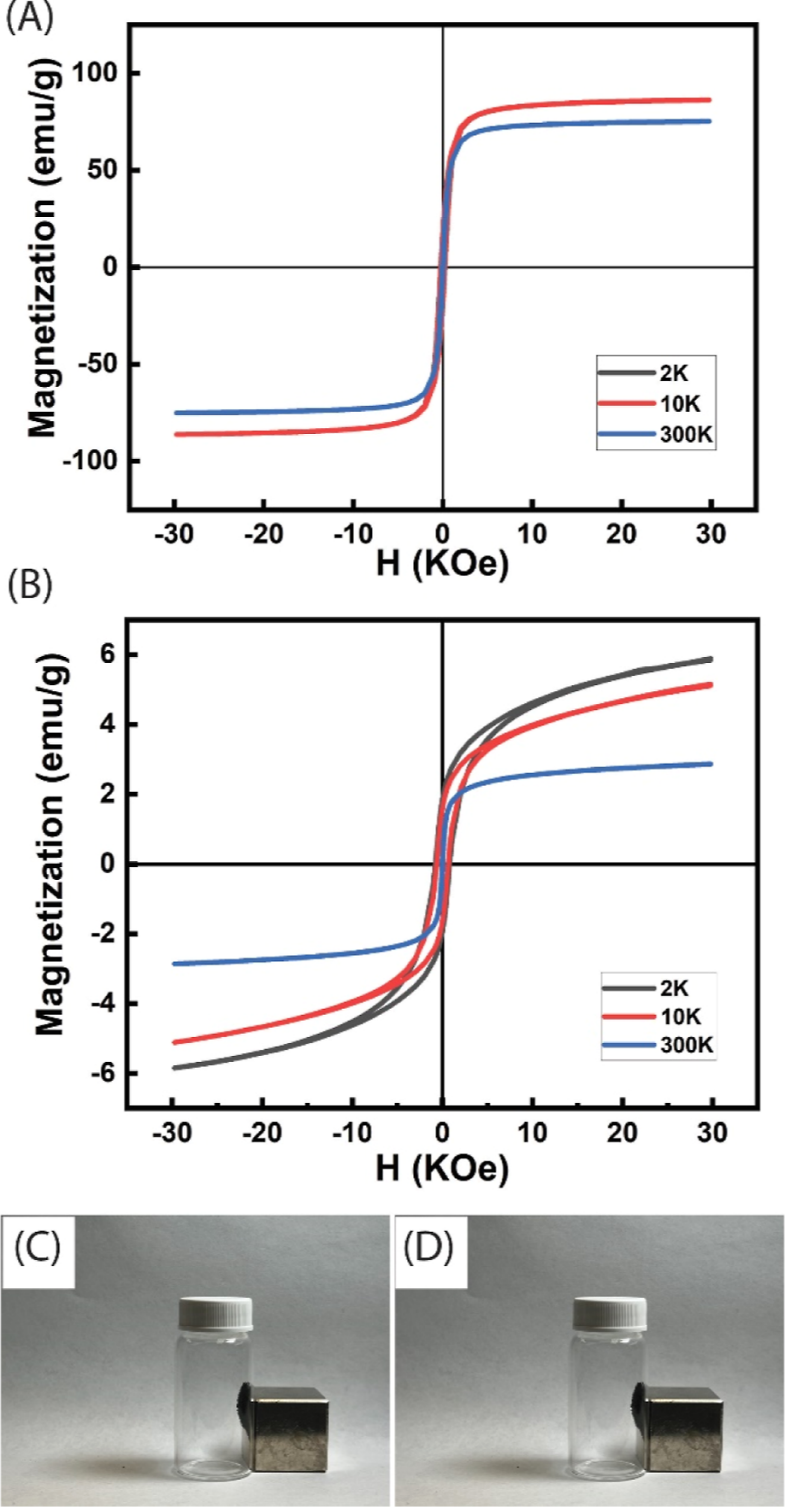
Magnetic hysteresis curve of (A) Fe_3_O_4_ and (B) Fe_3_O_4_/MCC/Co-ZIF-8. Photographs of the magnetic separation of (C) Fe_3_O_4_ and (D) Fe_3_O_4_/MCC/Co-ZIF-8 with a permanent magnet (photograph courtesy of A.M., Copyright 2024).

Figure 6 shows the magnetic hysteresis curve of Fe_3_O_4_ and Fe_3_O_4_/MCC/Co-doped ZIF-8 nanocomposites at 2, 10, and 300 K to study their magnetic behaviors under varying temperatures. The saturation magnetization is notably higher at 2 and 10 K compared to 300 K due to reduced thermal agitation at lower temperatures. The hysteresis loops of the Fe_3_O_4_ and Fe_3_O_4_/MCC/Co-doped ZIF-8 nanoparticles exhibit ferromagnetic behavior. The magnetization saturation of Fe_3_O_4_/MCC/Co-doped ZIF-8 is smaller than that of Fe_3_O_4_ nanoparticles due to the addition of nonmagnetic Co-doped ZIF-8 and MCC, which decreases the total magnetic moment in the sample. Additionally, as shown in Figure 6C,D, both Fe_3_O_4_ and Fe_3_O_4_/MCC/Co-ZIF-8 are magnetic and are attracted to an external magnet.

**Figure 7:**
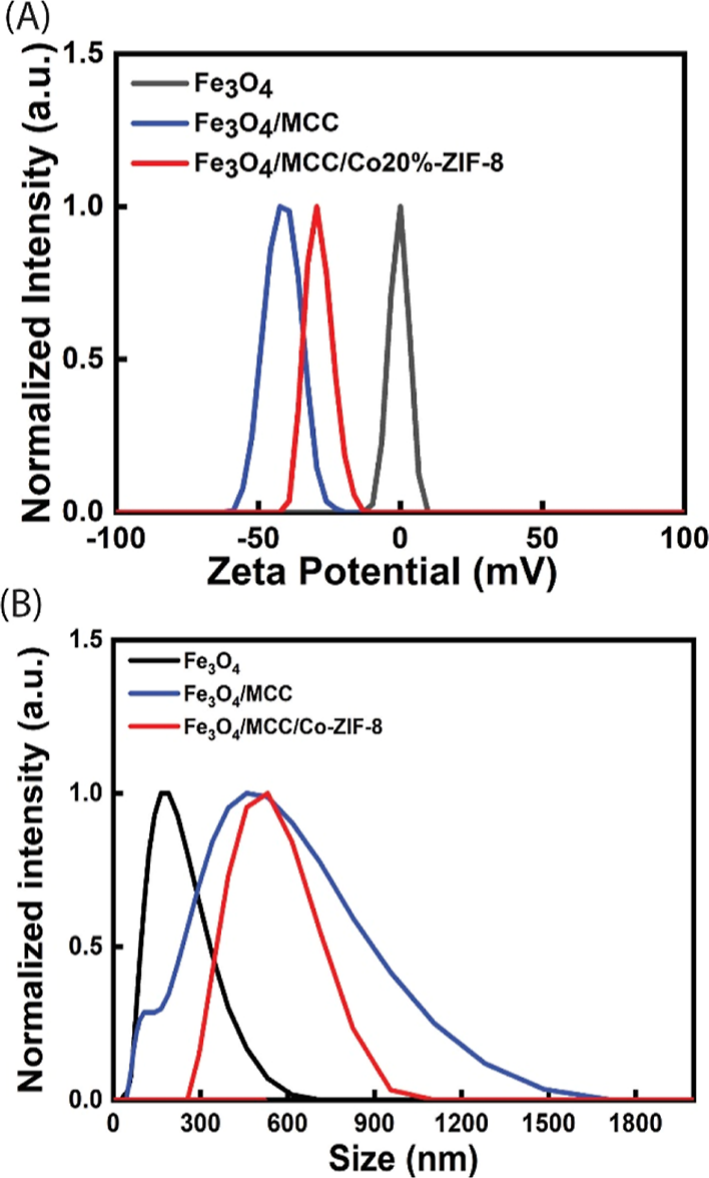
(A) ζ-Potential of Fe_3_O_4_, Fe_3_O_4_/MCC, and Fe_3_O_4_/MCC/Co-ZIF-8. (B) Size distribution of Fe_3_O_4_, Fe_3_O_4_/MCC, and Fe_3_O_4_/MCC/Co-ZIF-8.

DLS analysis was used to measure the zeta potential and size distribution of the magnetic samples. The zeta potential measurements displayed in Figure 7A confirm an increase in the magnitude of the negative zeta potential, indicating a strengthening of repulsive forces between particles, thereby reducing agglomeration. This validates the successful implementation of cellulose as a strategy to mitigate agglomeration. Furthermore, the functionalization of Fe_3_O_4_/MCC with Co-ZIF-8 does not result in a significant reduction of the zeta potential to lower values but maintains negative zeta potentials. These negative zeta potentials contribute to better dispersion of the photocatalyst in the photocatalytic reaction media via electrostatic repulsions. Figure 7B shows the plots of size distribution of Fe_3_O_4_ to Fe_3_O_4_/MCC and Fe_3_O_4_/MCC/Co-ZIF-8, with peaks around 160, 300, and 630 nm, respectively. This confirms that particle sizes increase when Fe_3_O_4_ is modified with MCC and increase further after modification with Co-ZIF-8. The broader peaks of Fe_3_O_4_/MCC and Fe_3_O_4_/MCC/Co-ZIF-8 compared to that of Fe_3_O_4_ indicate a wider range of particle sizes for these composite materials.

### 3.2 Photocatalytic Degradation of MB by Different Catalysts

**Figure 8:**
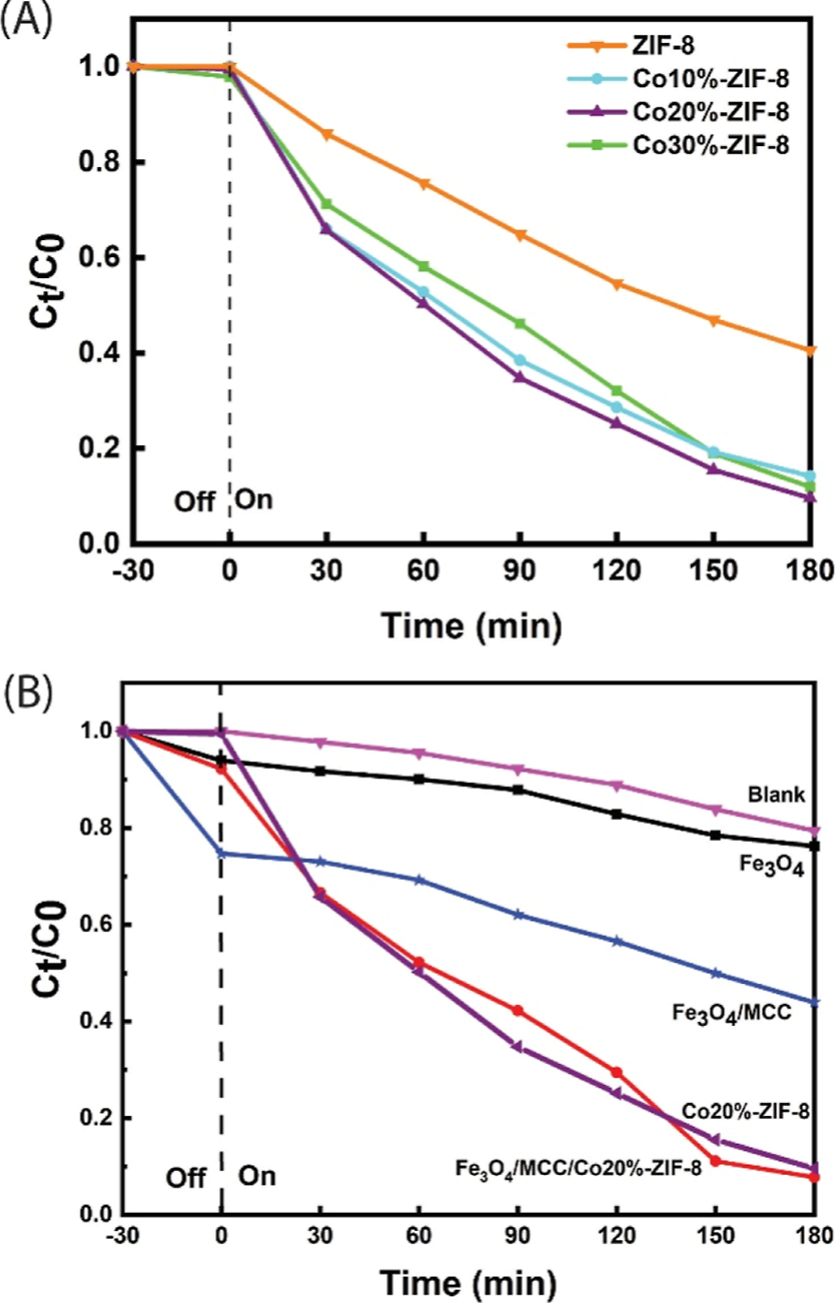
Photodegradation of MB under visible light irradiation by (a) different ZIF samples. (b) Different magnetic composites vs Co20%-ZIF-8.

The photodegradation of MB by a catalyst under visible light irradiation was examined, revealing the enhanced performance of Co-ZIF-8s compared with that of ZIF-8. The Co-ZIF-8 samples demonstrated superior activity, achieving a degradation rate of 91% within a 3 h time frame, whereas ZIF-8 achieved around 65% degradation within the same period, as shown in Figure 8A. This outcome verifies the success of our doping strategy in enhancing the photocatalytic activity of ZIF-8 under visible light. Among the Co-ZIF-8 samples, their photodegradation activities were generally comparable, with Co20%-ZIF-8 exhibiting slightly better results. Notably, Co20%-ZIF-8 displayed degradation rates of 75%, 91%, and 97.5% for MB within 120, 150, and 180 min, respectively. Consequently, the Co20%-ZIF-8 sample was chosen for the fabrication of the magnetic composite of Co-ZIF-8 (Figure 1). A control experiment was carried out to assess the self-sensitized photodegradation of MB without utilizing any catalyst (blank in Figure 8B), showing that the MB degradation under the same 20 W lamp irradiation over a 3 h period was negligible.

Fe_3_O_4_ exhibited low photodegradation activity due to its small band gap and rapid recombination of photogenerated charge carriers, consistent with reports from previous studies.^37^ The Fe_3_O_4_/MCC composite demonstrated notably higher MB removal in the dark compared to other samples. This outcome indicates that the primary mechanism behind MB removal by Fe_3_O_4_/MCC is adsorption, rather than photodegradation. The results of the zeta potential measurements showed that the surface of Fe_3_O_4_/MCC is negatively charged, facilitating the electrostatic interactions with the positively charged MB.^38^ Initially, there is rapid adsorption of MB by Fe_3_O_4_/MCC, but the rate of MB removal diminishes gradually as the adsorption sites on the surface of Fe_3_O_4_/MCC become saturated.

As previously mentioned, the photodegradation of MB by Co-doped ZIFs is superior to that of ZIF-8. Figure 8B demonstrates that the Fe_3_O_4_/MCC/Co20%-ZIF-8 composite also exhibits excellent photocatalytic activity, with MB photodegradation rates of 71%, 93%, and 97% within 90, 120, and 180 min, respectively. These findings confirm that the photocatalytic activity of Fe_3_O_4_/MCC/Co20%-ZIF-8 surpasses that of Fe_3_O_4_ and Fe_3_O_4_/MCC magnetic composite and is comparable to that of Co20%-ZIF8, indicating that Co20%-ZIF-8 retain its catalytic performance on the surface of the Fe_3_O_4_/MCC magnetic composite. This outcome is consistent with our characterization data, which show the preservation of ZIF crystallinity in the composite materials and demonstrate the success of our approach in enhancing the photocatalytic activity of Fe_3_O_4_ nanoparticles through the incorporation of Co20%-ZIF-8.

### 3.3 Radical Scavenger and Reusability Test

**Figure 9:**
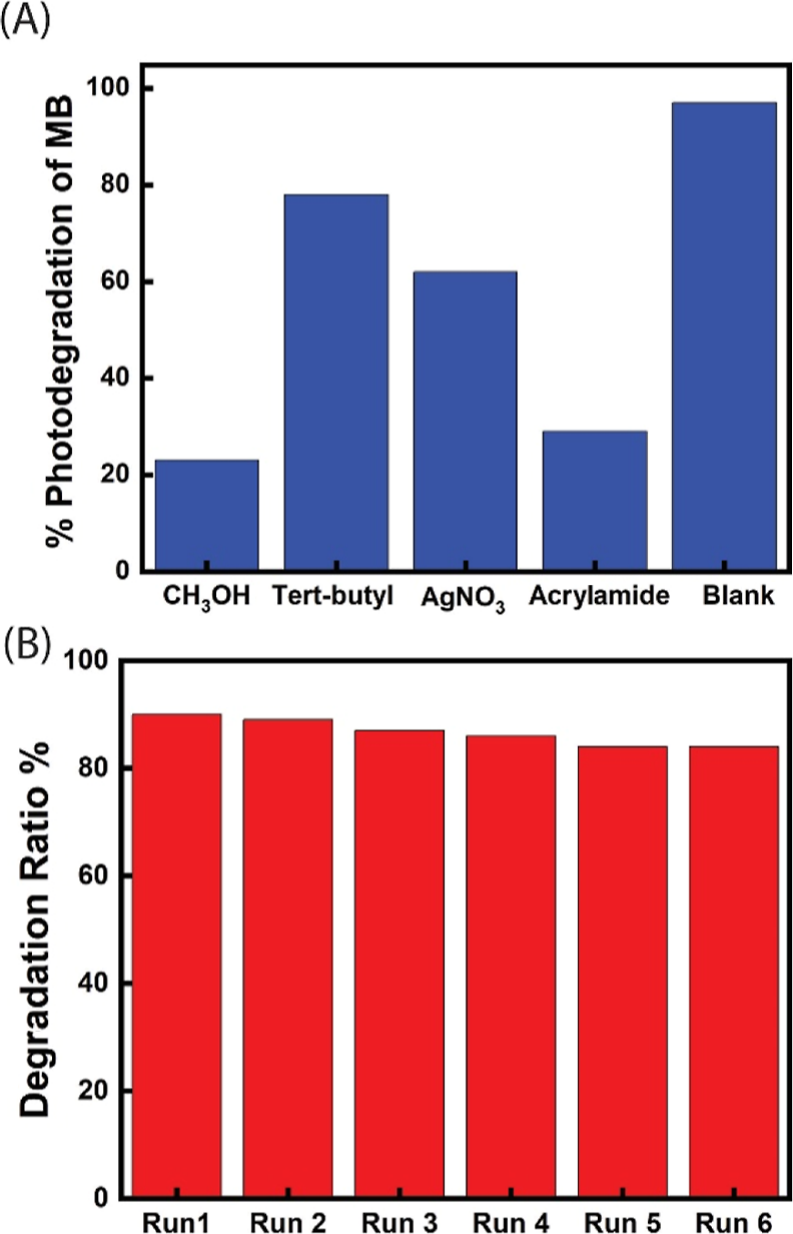
(A) Effects of different radical scavengers on the photodegradation of MB. (B) Photodegradation ratio of the catalyst at 180 min in six consecutive cycles.

Electron (e), hole (h^+^), superoxide radicals (O_2_^•–^), and hydroxyl radicals (^•^OH) are typical reactive species in the photocatalytic process. To investigate the photodegradation mechanism of MB by Fe_3_O_4_/MCC/Co20%-ZIF-8, radical scavengers were employed to trap potential photogenerated ROS. AgNO_3_, CH_3_OH, acrylamide, and *t*-butyl alcohol were used to scavenge the electron, hole, superoxide radicals, and hydroxyl radicals, respectively. As shown in Figure 9A, a significant decrease in the MB photodegradation was observed upon the application of hole and superoxide scavengers, suggesting that hole and superoxide radicals are the major ROS involved in the degradation of MB by the catalyst. Moreover, the reusability of the magnetic composite catalyst was tested by comparing the photocatalytic degradation of MB by Fe_3_O_4_/MCC/Co20%-ZIF-8 after six consecutive runs. The catalyst was recaptured from the reaction mixture readily using a magnet and washed before the next run. As shown in Figure 9B, the minimal decrease in photocatalytic degradation over six runs demonstrates the magnetic catalyst’s excellent reusability. The XRD (Figure S3) and SEM (Figure S4) of the recycled Fe_3_O_4_/MCC/Co20%-ZIF-8 composite confirmed that it retains the morphology and crystalline structure after 6 runs.

### 3.4 Band Structure of the Photocatalysts

**Figure 10:**
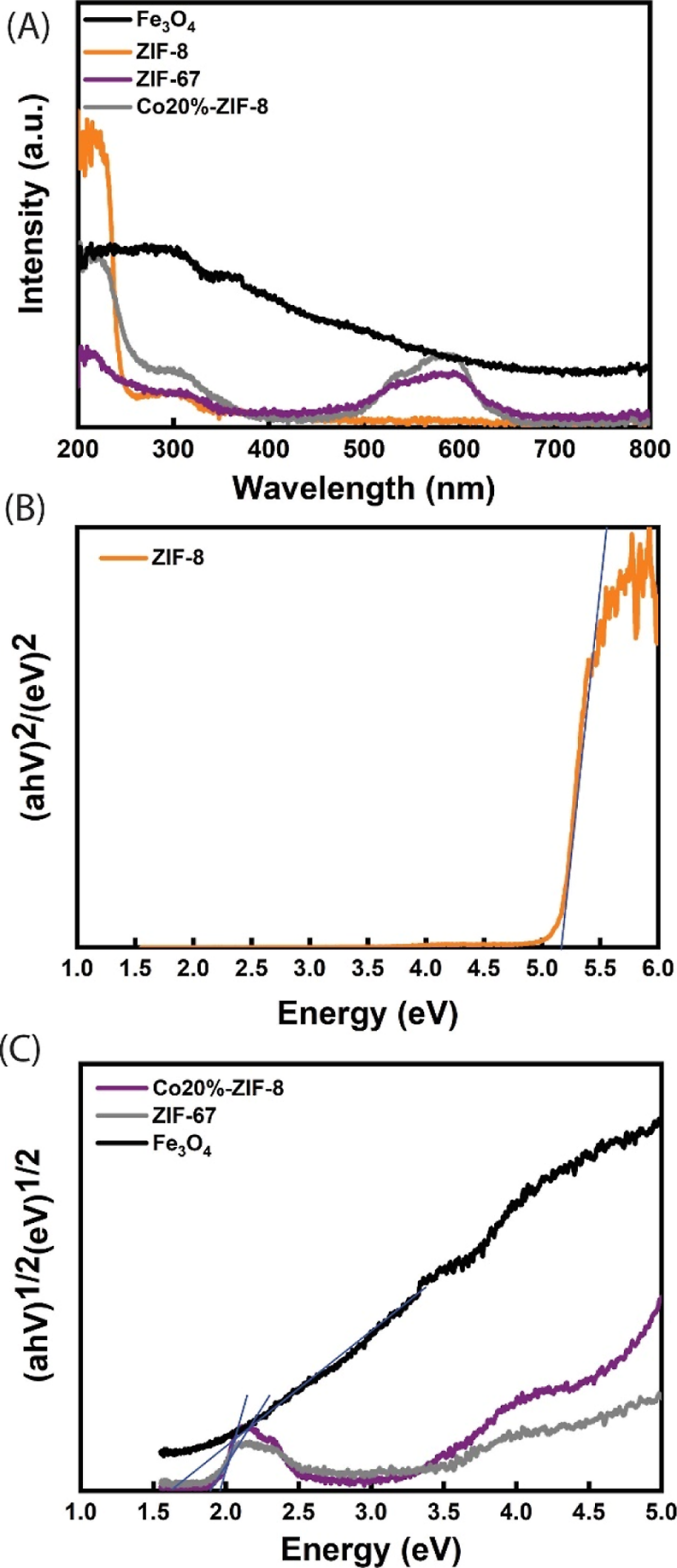
(A) UV–vis diffuse reflectance spectra of Fe_3_O_4_, ZIF-8, ZIF-67, and Co20%-ZIF-8. Tauc plots: (B) ZIF-8, (C) Fe_3_O_4_, ZIF-67, and Co20%-ZIF-8.

The optical properties of Fe_3_O_4_, ZIF-8, ZIF-67, and Co20%-ZIF-8 were studied using DRS, and the results are shown in Figure 10A. The DRS data were used to generate Tauc plots to estimate the band gaps of the mentioned samples. This analysis was performed to assess the effect of cobalt doping on the band gap of ZIF-8. Additionally, the band gap values along with the estimated valence band (VB) and conduction band positions were used to determine the band alignment between Fe_3_O_4_ and Co20%-ZIF-8, which is critical for explaining the mechanism of electron transfer within the composite. The band gaps were determined by plotting (α*h*ν)^2^ and (α*h*ν)^1/2^ for direct and indirect band gaps vs photon energies, followed by extrapolating the linear regions to the energy axis, as shown in Figure 10B,C, respectively.^39−41^ The calculated band gaps for ZIF-8, ZIF-67, and Co-ZIF-8 are 5.15, 1.89, and 1.98 eV, respectively. The results confirm that cobalt doping successfully decreases the band gap of ZIF-8, which could explain the enhanced photocatalytic activity of Co-ZIF-8 under visible light. The band gap of Fe_3_O_4_ was also calculated as 1.59 eV.

**Figure 11:**
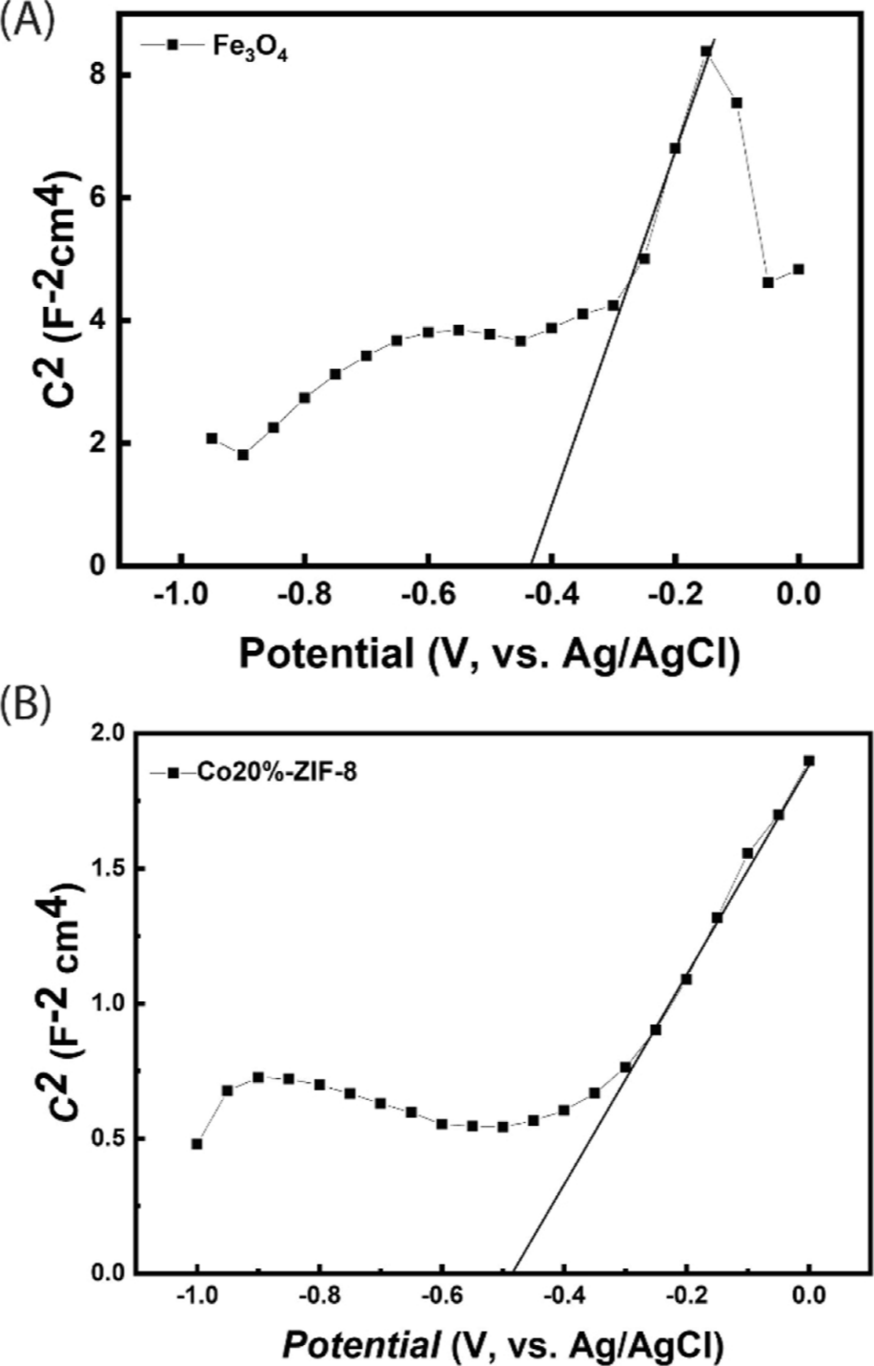
Mott–Schottky plots for (A) Fe_3_O_4_ and (B) Co20%-ZIF-8 electrodes.

Mott–Schottky plots were used to determine the flat bands and, consequently, the conduction bands (CB) of Fe_3_O_4_ and Co20%-ZIF-8. By knowing the band gap from DRS analysis, the VB of these materials can be calculated. So, the data from DRS and Mott–Schottky plots allow us to understand the energy levels and charge transport behavior in the investigated Fe_3_O_4_/MCC/Co20%-ZIF-8 composite. As shown in Figure 11, the positive slope of Mott–Schottky plots indicates that both Fe_3_O_4_ and Co20%-ZIF-8 are n-type semiconductors.^42^ The flat bands of both samples were estimated as −0.43 and −0.48 V versus Ag/AgCl (saturated KCl) and −0.23 and −0.28 V versus SHE, respectively.

According to the literature,^43^ the CB of n-type semiconductors are more negative by about 0.1 V than their flat potentials. Therefore, the CB of Fe_3_O_4_ and Co20%-ZIF-8 were estimated to be −0.33 and −0.38 V vs SHE, respectively. Given the band gaps of Fe_3_O_4_ (1.59 eV) and Co20%-ZIF-8 (1.98 eV), the corresponding VB potentials are 1.26 and 1.6 V vs SHE. The values of CB potentials are close to the redox potential of the O_2_/O_2_^•–^ couple (−0.33 V vs SHE), suggesting that the photocatalysts can transfer electrons to oxygen to produce the superoxide. This finding is consistent with the results of scavenger’s tests used to study the mechanism of photodegradation of MB, which showed that holes and superoxide radicals are the major ROS involved in the degradation process.

### 3.5 Rate Order of the Photocatalytic Degradation of MB by As-Prepared Samples

The kinetics of the photodegradation of MB using Fe_3_O_4_/MCC/Co20%-ZIF-8 as the photocatalyst were studied. By examining both pseudo-first- and pseudo-second-order kinetics, the order of the reaction was determined. For pseudo-first-order kinetics, ln (*C*_0_/*C*_*t*_) was plotted against time, while for pseudo-second-order kinetics, (1/*C*_*t*_ – 1/*C*_0_) was plotted against time. The obtained linear fitting correlation constants were 0.97 and 0.77 for Co20%-ZIF-8 in the pseudo-first-order and pseudo-second-order kinetics, respectively. Similarly, for Fe_3_O_4_/MCC/Co20%-ZIF-8, the correlation constants were found to be 0.92 and 0.68 for the pseudo-first-order and pseudo-second-order kinetics, respectively. These results indicate that the photocatalytic degradation kinetics of Fe_3_O_4_/MCC/Co20%-ZIF-8 follows a pseudo-first-order reaction.

The photodegradation rate constants for Co20%-ZIF-8 and Fe_3_O_4_/MCC/Co20%-ZIF-8 are 12.61 × 10^–3^ and 13.78 × 10^–3^ min^–1^, respectively. These findings demonstrate that the inclusion of the magnetic composite (Fe_3_O_4_/MCC) does not compromise the photocatalytic efficiency of the standalone Co-doped ZIF-8 (see Figure S1 in Supporting Information).

### 3.6 Photodegradation Mechanism

**Figure 12:**
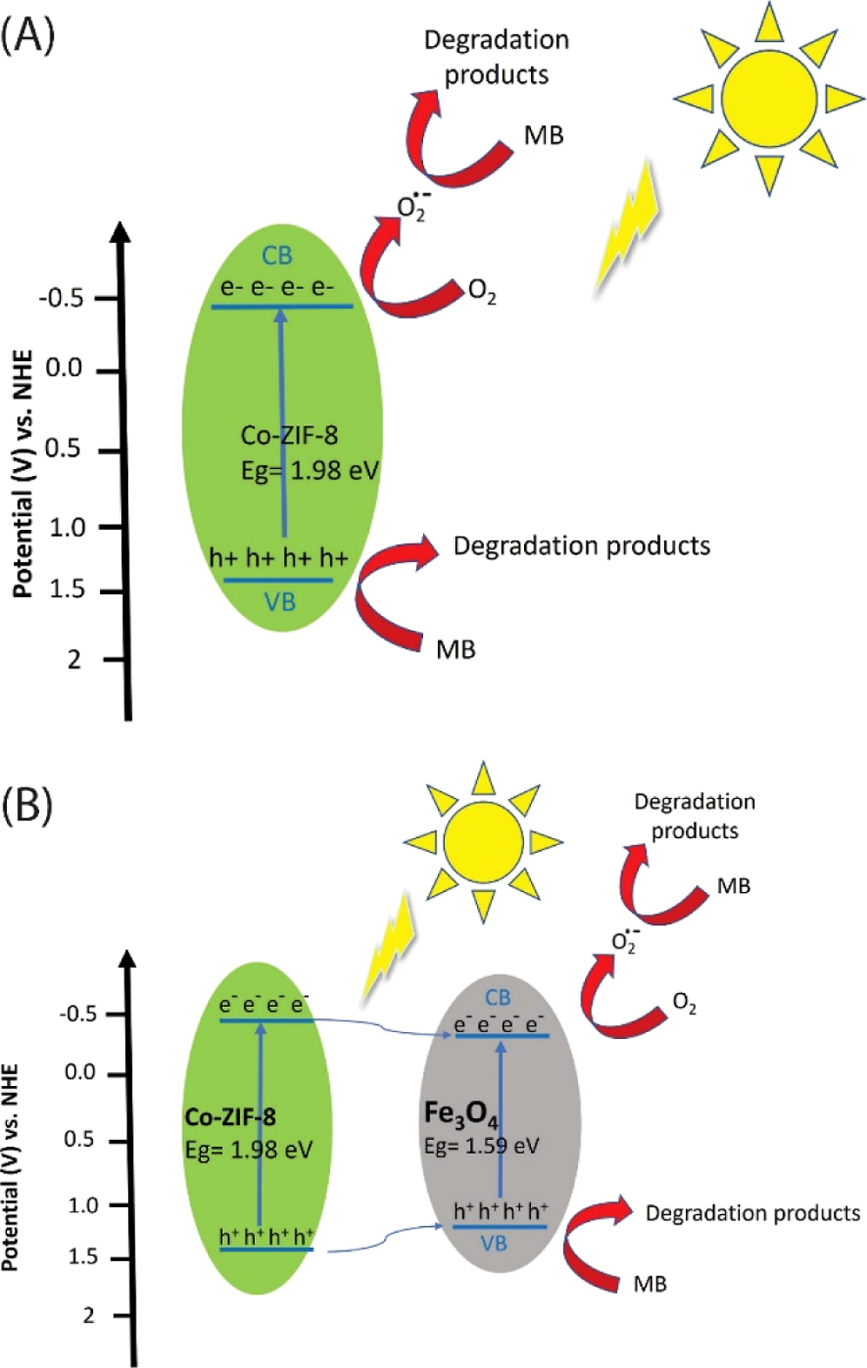
Schematic diagram of the proposed mechanism of photodegradation of MB by (A) Co-ZIF8 and (B) Fe_3_O_4_/MCC/Co20%-ZIF-8.

As mentioned earlier, the CB of Co-ZIF-8 is more negative than the redox potential of *E*^0^ (O_2_/O_2_^•–^) = −0.33 eV vs SHE. This allows Co-ZIF-8 to produce the radical superoxide (eq 2). Additionally, the photogenerated holes can be directly transferred to the MB (eq 1). As discussed earlier, Co(II) reduces the band gap and activates the ZIF-8 under visible light, while also acting as an electron-trapping agent to quench the recombination of photogenerated electrons and holes in Co-ZIF-8.^44^ The schematic of the proposed mechanism of Co20%-ZIF-8 is illustrated in Figure 12A.

Photocatalyst composites have different types of charge transfer based on their band alignment.^45,46^ In the Fe_3_O_4_/MCC/Co20%-ZIF-8 composite the band edges of the Fe_3_O_4_ contain those of Co-ZIF-8, and the system forms a type I heterojunction. In this heterojunction, both photogenerated electrons and holes are concentrated on the CB and VB of Fe_3_O_4_, respectively. Although Type I heterojunctions suffer from reduction on redox potential,^47^ this effect is not significant for the composite’s performance in MB removal. This is because the CB of Fe_3_O_4_ is close to the CB of Co-ZIF-8, as well as the oxygen reduction potential. Additionally, holes can also be transferred to the MB from the VB of Fe_3_O_4_ (eq 1). The Type 1 heterojunction promotes electron–hole separation and inhibits the recombination of photogenerated electrons and holes in Fe_3_O_4_.

The concentrated electrons on the CB band of Fe_3_O_4_ have a high enough reduction potential and reduce oxygen to radical superoxide (*E*^0^ (O_2_/O_2_^•–^) = −0.33 eV vs SHE), making superoxide the primary ROS for MB degradation.^48^ (eq 2). The oxidation potential of the h_VB_^+^ is not strong enough to oxidize OH^–^ and produce ^•^OH radicals (*E*^0^ (OH^–^/^•^OH) = 1.99 eV vs SHE).^48^ So, the degradation of MB by hydroxyl radicals is minor. According to scavenger experiments, direct oxidation of MB by holes is the second major degradation process after radical superoxide degradation (eqs 1 and 3). The schematic of the proposed mechanism of Fe_3_O_4_/MCC/Co20%-ZIF-8 is shown in Figure 12B.
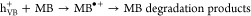
1
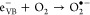
2
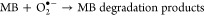
3

## 4 Conclusions

This study reports on a green, facile, fast, and low-cost method for synthesizing cobalt-doped ZIF-8s and their magnetic composites. The photocatalytic activity of the prepared composites was assessed by studying the photodegradation of MB under visible light irradiation using LEDs. The cobalt-doped ZIF-8 samples exhibited superior activity compared to ZIF-8 in the photodegradation of MB. The rate constants of photodegradation of MB by Co20%-ZIF-8 and Fe_3_O_4_/MCC/Co20%-ZIF-8 were found to be 12.61 × 10^–3^ min^–1^ and 13.78 × 10^–3^ min^–1^, respectively. These results confirm that coupling Co20%-ZIF-8 to magnetic nanoparticles did not decrease the photocatalytic activity. Scavenger tests and Mott–Schottky plots were used to investigate the mechanism of the photodegradation. These experiments revealed that both Co20%-ZIF-8 and Fe_3_O_4_/MCC/Co20%-ZIF-8 produce holes and superoxide as major ROS upon visible light irradiation. Fe_3_O_4_/MCC/Co20%-ZIF-8 was easily separated from the aqueous media by a magnet and could be recycled and reused for three consecutive cycles without a significant decrease in its photocatalytic activity.

## Supporting Information Available

Additional figures including kinetic model fittings for the photodegradation as discussed in the main text (PDF)

## Abbreviations

MOF: metal–organic frameworks
ZIF: zeolite imidazolate frameworks
MB: methylene blue
ROS: reactive oxygenated species
SOD: sodalite
2-MeIm: 2-methylimidazole
MCC: microcrystalline cellulose
FT-IR: Fourier-transform infrared spectroscopy
EIS: electrochemical impedance spectroscopy
WAXS: wide-angle X-ray spectroscopy
SEM: scanning electron microscopy
VB: valence band
CB: conduction band
