# Supplementary material for: Fast and Facile Synthesis of Cobalt-Doped ZIF-8 and Fe3O4/MCC/Cobalt-Doped ZIF-8 for the Photodegradation of Organic Dyes under Visible Light
Source: ACS Omega. 2024 Dec 7;9(50):49239–48. doi: 10.1021/acsomega.4c06142 (PMC11656232; doi:10.1021/acsomega.4c06142)
Supplement: Supplementary file 1 — ao4c06142_si_001.pdf [file ao4c06142_si_001.pdf]

Supporting Information

Fast and facile synthesis of cobalt doped ZIF-8 and Fe_3O_4/MCC/Cobalt doped-ZIF-8 for photodegradation of organic dyes under visible light

Amin Mehrehjedy,[a] Piyush Kumar,[a] Zachary Ahmad,[b] Penelope Jankoski,[b] Anuraj S. Kshirsagar,[c] Jason D. Azoulay,[d] Xuyang He,[e] Mahesh K. Gangishetty,[c,f] Tristan D. Clemons,[b] Xiaodan Gu,[b] Wujian Miao,[a] Song Guo[a]*

a. Department of Chemistry and Biochemistry, School of Mathematics and Natural Sciences, University of Southern Mississippi, Hattiesburg, MS, 39406, United States

b. School of Polymer Science and Engineering, The University of Southern Mississippi, Hattiesburg, MS 39406, United States

c. Department of Chemistry, Mississippi State University, Starkville, MS 39762, United States

d. School of Chemistry and Biochemistry and School of Materials Science and Engineering, Georgia Institute of Technology, Atlanta, GA 30332, United States

e. School of Criminal Justice, Forensic Science, and Security, The University of Southern Mississippi, Hattiesburg, Mississippi 39406, United States

f. Department of Physics and Astronomy, Mississippi State University, Mississippi State, Mississippi 39762, United States

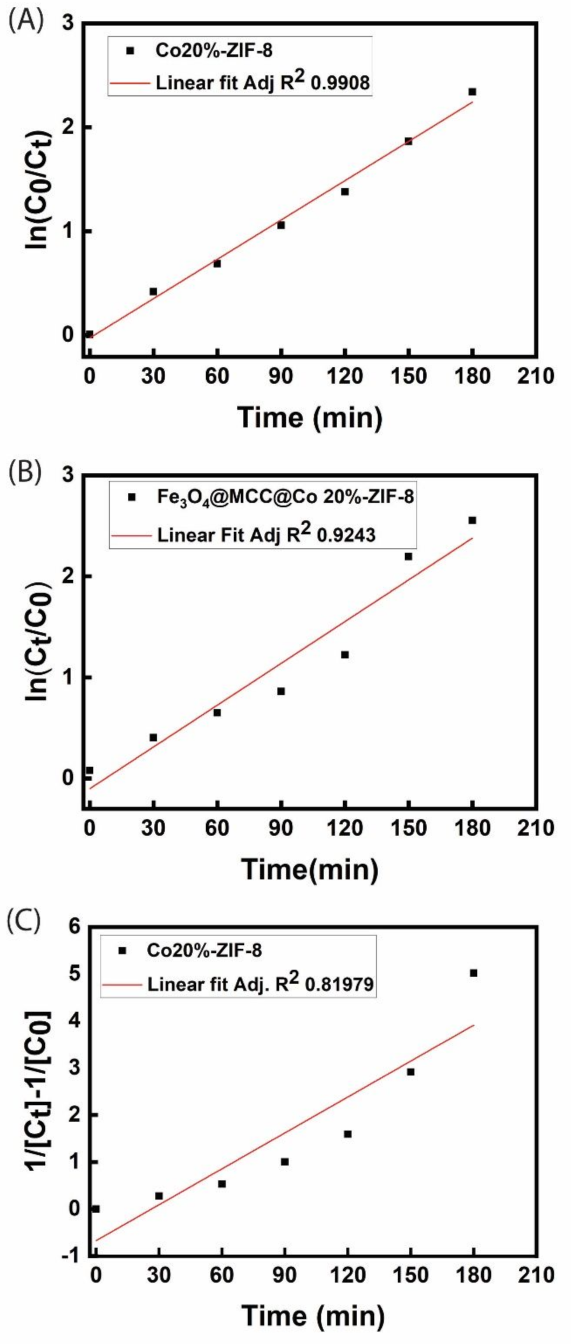

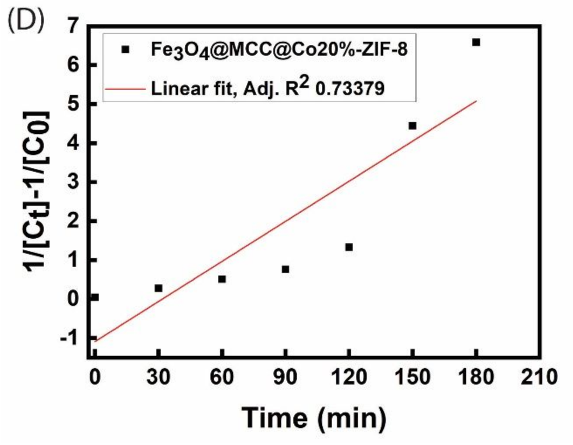

Figure S1. Kinetic models for the photodegradation of methylene blue (MB): Pseudo-first-order kinetic model for (A) Co20%-ZIF-8 and (B) Fe_3O_4@MCC@Co20-ZIF-8; Pseudo-second-order kinetic model for (C) Co20%-ZIF-8 and (D) Fe_3O_4@MCC@Co20-ZIF-8.

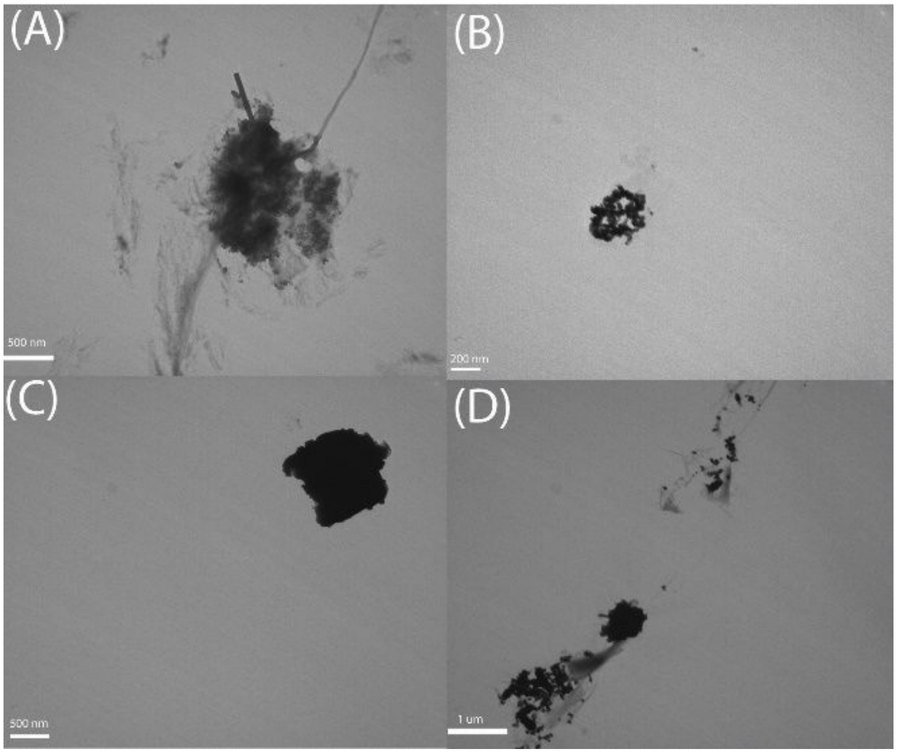

Figure S2. TEM of (A) Co20%-ZIF-8, (B) Fe_3O_4, (C) and (D) Fe_3O_4/MCC/Co-20%-ZIF-8.

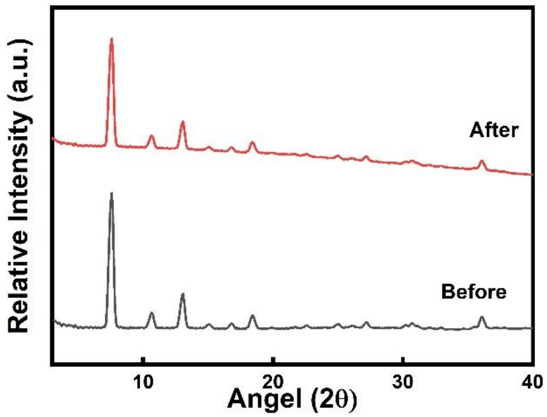

Figure S3. The wide angle X-ray scattering (WAXS) of Fe_3O_4/MCC/Co20%-ZIF-8 (Intensity × 5), Before and after reusability test.

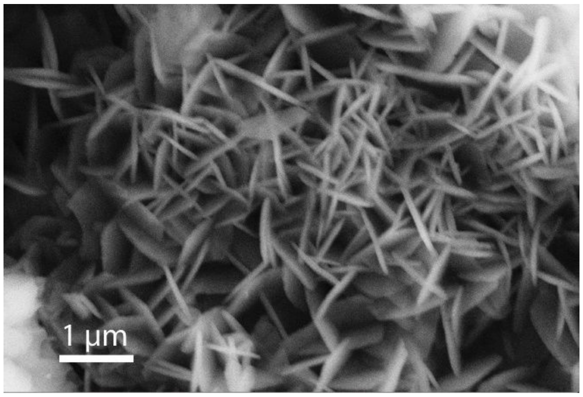

Figure S4. The SEM of Fe_3O_4/MCC/Co20%-ZIF-8 after reusability test.
